# Valemetostat–SAHA-Driven Acetylation of p53 via SET/TAF-Iβ Displacement and p300 Activation Modulates Cell Cycle Regulators in Pancreatic Cancer Cells

**DOI:** 10.3390/biomedicines13092279

**Published:** 2025-09-17

**Authors:** Michele Di Crosta, Francesca Chiara Ragone, Rossella Benedetti, Gabriella D’Orazi, Roberta Santarelli, Maria Saveria Gilardini Montani, Mara Cirone

**Affiliations:** 1 Department of Experimental Medicine, Sapienza University of Rome, 00161 Rome, Italy; michele.dicrosta@uniroma1.it (M.D.C.); ragone.1872024@studenti.uniroma1.it (F.C.R.); rossella.benedetti@uniroma1.it (R.B.); roberta.santarelli@uniroma1.it (R.S.); 2 Faculty of Medicine, UniCamillus—Saint Camillus International University of Health and Medical Sciences, 00131 Rome, Italy; gabriella.dorazi@unicamillus.org; 3 Unit of Cellular Networks and Molecular Therapeutic Targets, IRCCS Regina Elena National Cancer Institute, 00144 Rome, Italy

**Keywords:** pancreatic cancer, acetylation, mutp53, p21, CHK1, p300, SET/TAF-Iβ

## Abstract

**Background/Objective:** Aberrant acetylation and methylation of histone and non-histone proteins contribute to carcinogenesis. Among non-histone proteins, wild-type (wt) p53 is particularly notable for the critical role that acetylation and methylation play in regulating its stability and function. Although with opposite outcomes, these post-translational modifications (PTMs) can also affect mutant forms of p53 (mutp53), which are frequently detected in cancers. These proteins may acquire oncogenic properties, activating signaling pathways that promote carcinogenesis. Acetylation activates wtp53, while this PTM has been shown to destabilize mutp53, reducing cancer aggressiveness and improving the efficacy of anticancer therapies. In this study, we investigated the possibility of targeting mutp53 in pancreatic cancer cells by using a combination of EZH2 and HDAC inhibitors. **Methods:** Western blotting, qRT-PCR, and ChIP experiments were performed to address this question. **Results:** We found that the EZH2 inhibitor Valemetostat (DS) in combination with the histone deacetylase inhibitor SAHA displaced the SET/TAF-Iβ oncoprotein from mutp53 and increased its interaction with the acetyltransferase p300, which was responsible for p53 acetylation. Moreover, mutp53 was downregulated, p21 was upregulated, and CHK1 was reduced, increasing DNA damage and leading to a stronger impairment of pancreatic cancer cell survival compared with single-agent treatments. **Conclusions:** Our results reveal that combining epigenetic drugs such as Valemetostat and SAHA could be exploited to target mutp53 and improve the outcome of treatments for aggressive tumors harboring it, such as in pancreatic cancer.

## 1. Introduction

Genetic alterations such as mutations in oncogenes like *KRAS* and in tumor suppressors like *TP53* are frequently observed in pancreatic cancer and contribute to its aggressiveness [1]. However, epigenetic changes, including aberrant DNA methylation and acetylation and methylation of histones, also contribute to its onset and progression [2]. Unlike genetic mutations, epigenetic changes can be reversed by appropriate treatments, making them an especially promising area for therapeutic intervention. Among the drugs targeting epigenetic modifications are histone deacetylase inhibitors (HDACis) and histone methyltransferase inhibitors (KMTis), which are able to interfere with the activity of enzymes that transmit activating or repressive marks on histones [3], which may result in oncogene hyperexpression or tumor suppressor silencing [4]. HDACs are classified into four different classes: class I, comprising HDAC1, 2, 3, and 8; class IIa, comprising HDAC4 and 7; class IIb, which includes HDAC6 and 10; and class III, also called Sirtuins [4]. Inhibitors of HDACs show therapeutic potential against several types of cancers and, among other targets, they can downregulate mutp53. This is important because, aside from losing tumor suppressor function, mutp53 may acquire oncogenic properties and support carcinogenesis rather than counteracting it [5]. Previous studies have shown that treatment with the HDAC inhibitor (HDACi) SAHA induced the acetylation of heat shock protein 90 (HSP90), compromising its chaperoning function and destabilizing mutp53, which is known to be an HSP90 client [6].

However, acetylation may directly affect p53, both wtp53 and mutp53 proteins, since such a post-translational modification (PTM) rarely occurs in the DNA-binding domain (DBD), the site in which the protein is usually mutated [7]. Increased acetylation of mutp53 has been reported to promote its degradation [8,9]. In addition to acetylation, wtp53 may undergo lysine methylation [7,10], which favors its subsequent acetylation and results in its stabilization in response to DNA damage [11,12].

An acetylation–methylation interplay can occur also on histones, as for instance H3K27 acetylation by the acetyltransferase (HAT) p300 is influenced by tri-methylation of the same lysine residue mediated by the Enhancer of Zeste Homolog 2 (EZH2), a SET domain-containing methyltransferase, that works coordinately with other PcG proteins in the Polycomb Repressive Complex 2 (PRC2). p300 can also acetylate wtp53, leading to its transcriptional activation [13] and, interestingly, p53 can in turn activate p300 in a positive feedback loop [14,15]. It has been shown that p300 activity is sustained by SAHA, resulting, for example, in increased NF-kB acetylation [16]. Previous studies have shown that p300- or PCAF-mediated acetylation of histones, of wtp53 [17], and of Ku70/80 [18] is inhibited by SET/TAF-Iβ, a subunit of the Inhibitor of Histone Acetyltransferases (INHATs), a histone co-chaperone regulated by EZH2 [19]. The impact of the combination of EZH2 and HDAC inhibitors on mutp53 acetylation and stability has not been previously studied. In the present study, we evaluated whether the combination of the EZH2 methyltransferase inhibitor, Valemetostat, and the HDAC inhibitor, SAHA, could reduce the interaction of mutp53 with INHAT while enhancing that with p300, resulting in increased acetylation and destabilization of mutp53 in pancreatic cancer cells harboring various p53 mutations. We also investigated whether this combined treatment could modulate cell cycle regulators, increase DNA damage, and more significantly impair pancreatic cancer cell survival than treatment with SAHA or Valemetostat alone.

## 2. Materials and Methods

### 2.1. Cell Cultures and Treatments

Human pancreatic cancer cell lines PaCa44 and PT45 and Panc-1, obtained from Dr. M. von Bülow (University of Mainz, Mainz, Germany), Dr. H. Kalthoff (University of Kiel, Kiel, Germany), and the American Type Culture Collection (Rockville, MD, USA), were cultured as previously reported [8]. First, 2 × 10^5^ cells/well were seeded into 6-well plates in complete medium and treated for 48 h with the HDAC inhibitor Vorinostat (SAHA) (2.5 μM) (MedChemExpress, New York, NJ, 08852, USA, cat. HY-10221/CS-0589), the EZH2 methyltransferase inhibitor Valemetostat (DS-3201) (5 μM) (Selleckchem, Cologne, Germany, cat. S8926), or a combination of both. In some experiments, cells were pre-treated for 60 min with the p300 inhibitor A-485 (p300 inh) (100 μM) (Selleckchem, Cologne, Germany, cat. S8740) before exposure to SAHA/DS. In some experiments, cells were pre-treated with pifithrin (pif-α (30 μM) (Sigma-Aldrich, St. Louis, MO, USA, cat. P4359) for 30 min before exposure to SAHA/DS. In some experiments, 5-Azacitidine (5-AZA) (40 nM) (Selleckchem, Cologne, Germany, cat. S1782) was added every 24 h to SAHA/DS-3201-treated cells.

### 2.2. Chromatin Immunoprecipitation (ChIP) Assay

For the ChIP assay, approximately 5 × 10^5^ cells/mL, washed twice with PBS at room temperature, were used. Living cells were treated with formaldehyde (Sigma-Aldrich, St. Louis, MO, USA, cat. 252549) at final concentration of 1% to cross- link DNA and proteins. After 10 min, glycine (Sigma-Aldrich, St. Louis, MO, USA, cat. 68898), at a final concentration of 0.125 M, was added to stop the reaction. Then, cells washed in PBS were resuspended in 6 mL Lysis Buffer (Santa Cruz Biotechnology Inc., Dallas, TX, USA, cat n.sc-45000). The nuclear extract was collected by centrifugation at 2500 rpm for 5 min and washed in PBS. The nuclei were resuspended in 1.9 mL Lysis Buffer High Salt (Santa Cruz Biotechnology Inc., Dallas, TX, USA, cat n.sc-45001) and sonicated on ice in continuous mode for 1 min 4 times every 30 s to generate DNA fragments of size ranging from 2000 to 100 bp, with the majority of them ranging between 300 and 500 bp. The nuclear extract was then centrifugated for 15 min at 10,000 rpm at 4 °C. For IP, 500 µg of supernatant, precleared by incubation for 1 h at 4 °C with 50 μL of protein A-G plus agarose beads (Santa Cruz Biotechnology Inc., Dallas, TX, USA, cat n.sc-2003), was incubated overnight at 4 °C with monoclonal anti-p53 (DO-1) (Santa Cruz Biotechnology Inc., Dallas, TX, USA, cat n. sc-126). An amount of 50 μL of Protein A/G plus agarose was then added to the nuclear extract for 1 h at 4 °C. After centrifugation, the beads were washed with 1 mL Lysis Buffer High Salt then washed four times with Wash Buffer (Santa Cruz Biotechnology Inc., Dallas, TX, USA, cat n.sc-45003) and resuspended in Elution buffer. Reverse cross-linking was performed by adding Proteinase K (20 mg/mL) (Sigma-Aldrich, St. Louis, MO, USA, cat.03115887001) to 100 μL of the negative control or IP sample. The de-cross-linked samples were cleaned up with the Wizard DNA Clean-UP System (Promega, Madison, Wisconsin, USA, cat n. A7280). The p53 binding to p21 was evaluated by qRT-PCR using specific primers for the p21 promoter region, forward 5′-CCGCTCGAGCCCTGTCGCAAGGATCC-3′ reverse 5′-GGGAGGAAGGGGATGGTAG-3′, and the ChIP results were normalized with p21 present in the input of each immunoprecipitation.

### 2.3. Immunoprecipitation Assay

First, 1.5 × 10^6^ cells were treated with 2.5 μM SAHA and 5 μM DS-3201, singly or in combinations, for 48 h, and the immunoprecipitation (IP) assay was performed as previously reported [8]. For IP, he following antibodies were used: mouse anti-p53 and mouse anti-Ac-Lysine antibodies (Santa Cruz Biotechnology Inc., Dallas, TX, USA, cat. n. sc-126 and sc-32268). Precipitated proteins, obtained after centrifugation, were analyzed by Western blotting.

### 2.4. Western Blotting Analysis

After the treatments, the cells were centrifuged and lysed, and the released proteins were subjected to electrophoresis and transferred to nitrocellulose membranes (Bio-Rad, Hercules, CA, USA) as previously reported [20]. Blots were subsequently washed, incubated with specific HRP-conjugated secondary antibody, and subjected to ECL (Advansta, San Jose, CA, USA, #12045-D20).

### 2.5. Antibodies for Western Blotting Analysis

The following antibodies were used: rabbit polyclonal anti-acetylated Histone H3 (1:500) (Invitrogen, Waltham, MA, USA, PA5-114693), rabbit monoclonal anti-Histone H3 (1:4000) (Cell Signaling, Danvers, MA, USA, cat n. 4499T), mouse monoclonal anti-caspase3 (1:300) (Santa Cruz Biotechnology Inc., Dallas, TX, USA, cat n. sc-56053), rabbit polyclonal anti-Acetyl-p53 (Lys373; Lys382) (1:500) (Merckmillipore, Darmstadt, Germany, 06-758), mouse monoclonal anti-p53 (1:500) (Santa Cruz Biotechnology Inc., Dallas, TX, USA, cat n. sc-126), rabbit monoclonal anti-p21 (1:500) (Cell Signaling, Danvers, MA, USA, cat n. 2947T), SET/TAF1 (1:1000) (Proteintech, Manchester, UK, cat. n.55201-1-AP), CHK1 (1:500) (Santa Cruz Biotechnology Inc., Dallas, TX, USA, cat n. sc-8408), p300 (1:300) (Santa Cruz Biotechnology Inc., Dallas, TX, USA, cat n. sc-48343), and mouse monoclonal anti-γH2AX (1:100) (Santa Cruz Biotechnology Inc., Dallas, TX, USA, cat n. sc-517348). Mouse monoclonal anti-βActin (1:10,000) (Sigma-Aldrich, St. Louis, MO, USA, cat n. A5441) and mouse monoclonal anti-GAPDH (1:10,000) (Santa Cruz Biotechnology Inc., Dallas, TX, USA, cat n. A5316) were used to detect the loading control.

### 2.6. Densitometric Analysis

Image J software (1.47 version, NIH, Bethesda, MD, USA, http://imagej.nih.gov, downloaded on 10 February 2022) was used to quantify the proteins detected by Western blotting.

### 2.7. RNA Isolation and Quantitative Real-Time PCR Analysis

TRIzol-Reagent (Invitrogen, Carlsbad, CA, USA) was used to extract total RNA according to the manufacturer’s instructions. PCR analyses were performed using the following specific oligonucleotides:

P21 forw:5′-TACATCTCCCATTTCACCTAC-3′;
P21 rev: 5′CGTAATTTCTCCAAGATCTCC-3′;
ACT forw: 5′-TCACCCACACTGTGCCATCTACGA-3′;
ACT rev: 5′-CAGCGGAACCGCTCATTGCCAATGG-3′.

Transcripts were measured by real-time PCR using the SYBR Green assay (Applied Biosystems, Carlsbad, CA, USA) with a StepOne instrument and 7500 Fast Real-Time PCR System (Applied Biosystems). The PCR protocol was set at 95 °C—2 min, and the steps were repeated 40 times: (95 °C—10 s; 55 °C—20 s; 72 °C—1 s) and then 4 °C infinite. mRNA expression was quantified with the 2^−ΔΔCT^ method using *β-actin* gene expression as endogenous controls to compare mRNA expression. All samples were run in triplicate.

### 2.8. Transfection with p53 K382R Vector

To investigate the impact of 381/382 lysine acetylation in p53 changes observed in response to SAHA/DS treatment, cells were transfected with the pcDNA3-empty vector (EV) or p53K382R vector (in which lysine 381 and 382 were mutated to arginine to render p53 non-acetylatable at these residues) [21] by using Lipofectamine 2000 (Thermo Fisher Scientific, Waltham, MA, USA, 11668-027), as previously reported [8]. After 24 h, cells were treated or not with the SAHA/DS combination and collected for Western blotting analysis following 48 h of treatment.

### 2.9. Cell Assay Viability

The percentage of viable cells was obtained by counting the amount of Trypan blue (Sigma-Aldrich, Burlington, MA, USA, 72571) stained (dead) and unstained (live) cells by a Leitz Labovert FS Inverted Microscope.

### 2.10. Colony Forming Assays

Cells treated as above for 24 h were plated in 60 mm Petri dishes at a low density. After 12 days, crystal violet (0.5% in methanol) (Sigma-Aldrich) was added to the growing colonies to allow the analysis by using Image J, as previously reported [20].

### 2.11. Cell Cycle Analysis

The DNA content of the PaCa44 cell line treated as above and stained by Propidium Iodide (Sigma Aldrich, St Louis, MO, USA; #P4170) was analyzed by flow cytometry (FACScan, Becton Dickinson, Lincoln Park, NJ, USA) by using Cell Quest software (Version 6.0).

**Figure 1:**
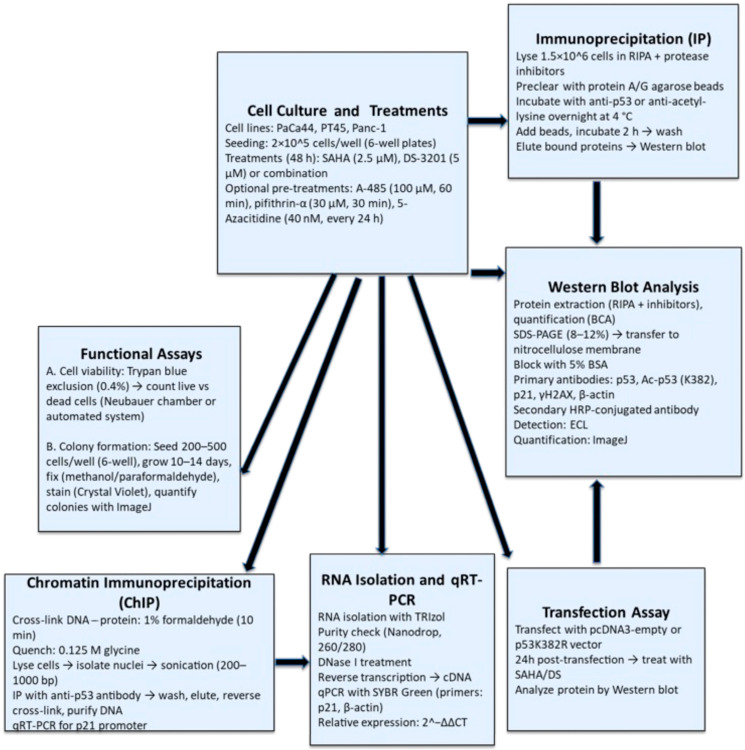
Flowchart summarizing the applied methodology.

The methodology is summarized in the following flowchart (Figure 1).

### 2.12. Statistical Analysis

Statistical analysis was performed by Graphpad Prism^®^ software (version 9; Graphpad Software Inc., La Jolla, CA, USA) using the Student’s *t*-test or a nonparametric one-way ANOVA to compare the data represented as histograms of mean value plus standard deviation (S.D.) of at least three independent experiments. The difference was considered statistically significant when the *p*-values were as follows: * < 0.05; ** < 0.01; *** < 0.001; and **** < 0.0001.

## 3. Results

### 3.1. Valemetostat in Combination with SAHA Increases Lysine 373/382 Acetylation of p53 and Upregulates p21

**Figure 2:**
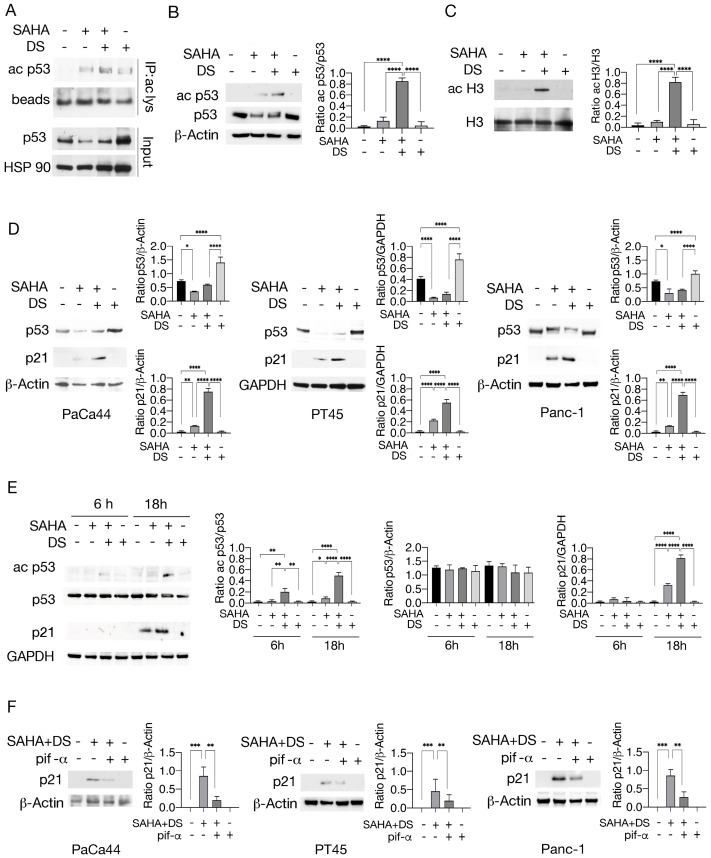
p53 acetylation and p21 expression are upregulated by SAHA/DS treatment in pancreatic cancer cells. PaCa44 pancreatic cancer cells were exposed for 48 h to SAHA (2.5 µM), DS (5 µM), or SAHA/DS combination (2.5 µM/5 µM M) or left untreated (CT). (**A**) Acetylation of p53 was evaluated by immunoprecipitation with anti-acetyl-lysine antibody and blotting with p53. Beads are shown as loading control. (**B**) Acetylation of p53 (ac p53) and total p53 expression was also evaluated by Western blotting by using anti-373/382 acetyl p53 and anti-p53 antibody. β-Actin was used as loading control. (**C**) Acetyl-histone H3 (ac H3) expression level was assessed by Western blotting in PaCa44 cells. (**D**) p53 and p21 expression as evaluated in PaCa44, PT45, and Panc-1 undergoing treatment by SAHA (2.5 µM), DS (5 µM), or SAHA/DS combination. β-Actin or GAPDH were used as controls. (**E**) Ac p53 and p21 time-course expression as evaluated by Western blotting analysis in PaCa44 cell line (**F**) p21 expression level as evaluated by Western blotting analysis in PaCa44, PT45, and Panc-1 following treatment by SAHA/DS in presence or in absence of pifithrin-α. β-Actin was used as loading control. The histograms indicate the mean plus SD of the densitometric analysis of three experiments and expressed as the ratio between molecules of interest and loading controls; *p*-value * < 0.05; ** < 0.01; *** < 0.001; and **** < 0.0001, as calculated by ANOVA test.

Since protein acetylation can be influenced by methyltransferase treatment and acetylation has been reported to regulate p53, in this study, we explored whether the combination of the EZH2 inhibitor Valemetostat (DS) with the HDAC inhibitor SAHA could result in increased p53 acetylation in pancreatic cancer cells. As shown in Figure 2A, DS, used at 5 μM, in combination with SAHA, used at 2.5 μM, increased the expression level of acetylated p53 in PaCa44, pancreatic cancer cells carrying the p53 C176S mutation, whereas SAHA alone slightly induced this effect. We then investigated whether acetylation could occur at lysine 373/382, since these residues on wtp53 have been reported to be deacetylated by HDAC6, which is inhibited by SAHA [22]. Using a specific antibody, we observed that p53 was acetylated at lysine 373/382 following treatment with SAHA/DS (Figure 2B). Histone H3 was also hyper-acetylated (Figure 2C), suggesting a broad acetylating effect induced by this treatment. Using the DO-1 antibody, which recognizes both wt and mutp53, we then found that mutp53 was downregulated by SAHA and slightly less by SAHA/DS in Paca44. This effect was also observed in PT45, a pancreatic cancer cell line harboring mutp53 R280K, and in Panc-1 cells harboring mutp53 R273H (Figure 2D). Because different forms of mutp53 can be regulated differently by the same treatments, and, although our results suggest that SAHA and SAHA/DS were capable of acting on different types of mutp53, the effect on other mutp53 remains to be studied. Interestingly, SAHA/DS treatment upregulated p21 in all cell lines tested, whereas SAHA alone was less effective in increasing its expression (Figure 2D). These effects were induced after 18 h of SAHA/DS treatment, while they were not evident after 6 h in PaCa44 cells (Figure 2E). Interestingly, the wtp53 inhibitor pifithrin-α counteracted the SAHA/DS- induced p21 upregulation (Figure 2F), suggesting that such treatment could reactivate wtp53, as reported in previous studies on prostate cancer cells where mutp53 acetylation restored wtp53 function [23]. This may also be suggested by the slight increase in p53 expression detected by the DO-1 antibody after SAHA/DS treatment. However, mutp53 acetylation could also enhance the activity of transcriptional coactivators such as p63 or p73, able to activate p21 expression [24] and susceptible to the inhibitory effect of pifithrin-α.

**Figure 3:**
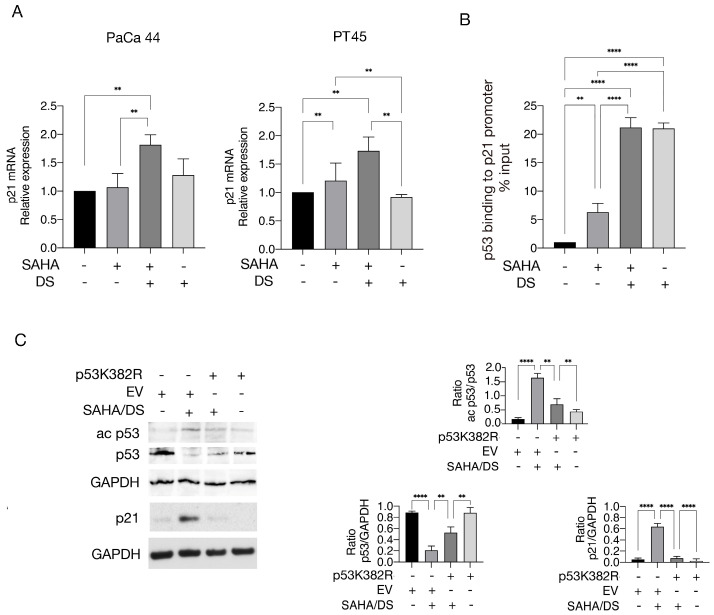
p21 is upregulated at transcriptional level by SAHA/DS combination. (**A**) *p21* mRNA expression in cells treated by SAHA, DS, or SAHA/DS as evaluated by q-RT PCR. (**B**) CHIP assay performed to investigate binding of p53 to *p21* promoter in cells treated by SAHA, DS, or combination of both by using DOI antibody. (**C**) Acetylation of p53 and p21 expression level in Paca44 cells following transfection with p53 K382R vector (+) or with empty vector (−). GAPDH was used as loading control. Histograms indicate the mean plus SD of the densitometric analysis out of three experiments and expressed as ratio between acetyl p53/p53, p53/GAPDH and p21/GAPDH. *p*-value ** < 0.01; and **** < 0.0001, as calculated by ANOVA test.

*p21* mRNA expression was also increased following SAHA/DS treatment (Figure 3A), indicating transcriptional regulation of this protein. We next assessed p53 binding to the *p21* promoter in cells exposed to SAHA, DS, or a combination of them by performing a ChIP assay. To this end, using a DOI antibody, we found that p53 binding to the *p21* promoter increased following treatment with DS and SAHA/DS and slightly with SAHA (Figure 3B). However, since *p21* was upregulated only with the SAHA/DS combination, this suggests that EZH2 inhibition could remove the repressive mark H3K27me3 from the *p21* promoter and allow p53 binding, but inhibition of HDAC activity was required for *p21* transcription. We then evaluated whether p53 acetylation could contribute to the increased p21 expression following SAHA/DS treatment. Toward this aim, we transfected PaCa44 cells with non-acetylatable p53 K382R mutant plasmid (Figure 3C), a plasmid in which lysines 381/382 are substituted by arginine to render the protein no longer acetylatable at these residues [21]. We found that p53 acetylation and downregulation were reduced following plasmid transfection, and p21 upregulation was also counteracted (Figure 3C). Overall, these findings suggest that EZH2 inhibition could allow p53 to bind to the promoter of *p21*, but acetylation was also required to activate *p21* transcription.

### 3.2. Valemetostat–SAHA Combination Enhances p53 Acetylation by Increasing Its Interaction with p300 While Reducing That with the Cochaperone SET/TAF-Iβ

**Figure 4:**
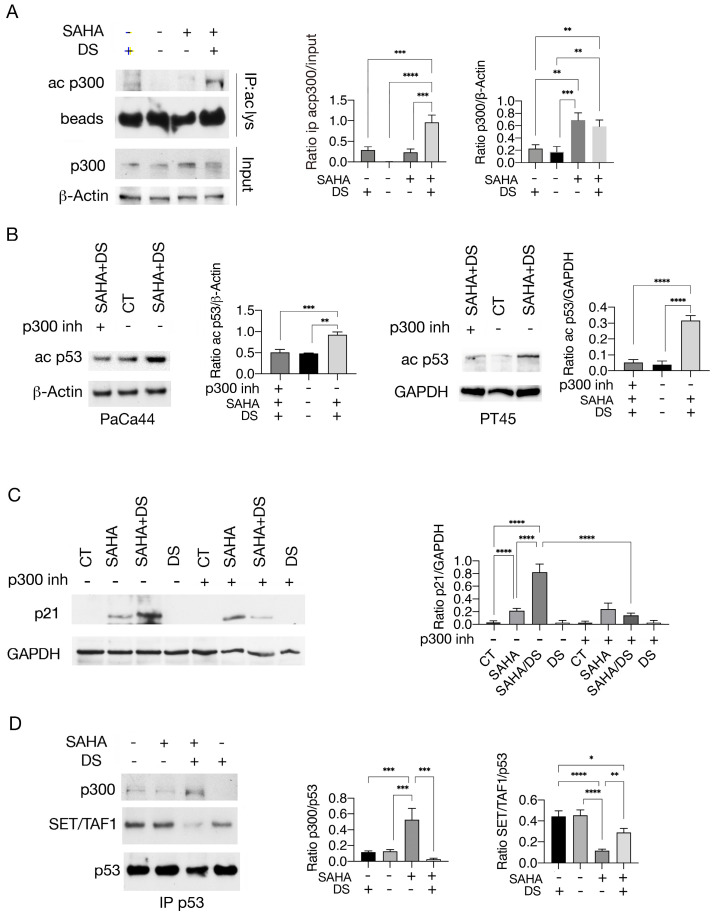
p53 acetylation in response to SAHA/DS treatment correlates with changes in its interaction with p300 acetyltransferase and the cochaperone SET/TAF-Iβ. (**A**) Acetylation of p300 was investigated by immunoprecipitation with anti-acetyl-lysine antibody and blotting with an anti-p300 antibody in PaCa44 cancer cells untreated (CT) or undergoing SAHA or SAHA/DS treatment. Beads are shown as loading control. (**B**) Acetyl-p53 (ac p53) as evaluated by Western blotting in PaCa44 and PT45 cells pre-treated (+) or not (−) by p300 inhibitor (p300 inh) and then treated with SAHA/DS or left untreated (CT). β-Actin and GAPDH represent loading controls. (**C**) p21 expression level as evaluated in PaCa44 cancer cells pre-treated (+) or not (−) with p300 inhibitor and exposed to SAHA, SAHA/DS, and DS or untreated control (CT). GAPDH was used as loading control. (**D**) Interaction of mutp53 with p300 and SET/TAF1β investigated by immunoprecipitation in PaCa44 cells treated with SAHA, SAHA/DS, and DS. P53 is shown as loading control. Histograms represent mean plus SD of densitometric analysis from three experiments and expressed as ratio between ac p300/beads and p300/β-Actin (**A**), ac p53/β-Actin and ac p53/GAPDH (**B**), p21/GAPDH (**C**), and p300/p53 and SET/TAF1/p53 (**D**). *p*-value * < 0.05; ** < 0.01; *** < 0.001; and **** < 0.0001 as calculated by ANOVA test.

Pan-HDAC inhibitors have been shown to stabilize the HAT p300 [25] and the inhibition of HDAC6, which can be mediated by SAHA, can potentiate this effect to the extent that its therapeutic focus shifts from the inhibition of HDAC6 itself to the stabilization of p300 [26]. It is known that p300 is involved in p53 acetylation [13] and can undergo autoacetylation upon activation [14]. Here, we observed that p300 was more acetylated following treatment with SAHA/DS compared to SAHA alone (Figure 4A), suggesting that it was activated following this drug combination. To investigate whether p300 could play a role in p53 acetylation in SAHA/DS-treated cells, we used the p300 inhibitor A485 (p300 inh). As shown in Figure 4B, 373/382 lysine acetylation of p53 was reduced in the presence of this inhibitor, indicating that p300 played a key role in p53 acetylation induced by SAHA/DS treatment. Interestingly, p21 upregulation was also strongly reduced by the p300 inhibitor in SAHA/DS-treated cells (Figure 4C).

Next, we investigated the molecular mechanisms leading to increased p53 acetylation in response to SAHA/DS treatment. Previous studies reported that the histone cochaperone SET/TAF-Iβ binding to wtp53 [17] or to Ku70/80 [27] could inhibit their p300- or PCAF-mediated acetylation. Here, we investigated whether SET/TAF-Iβ could be basally bound to mutp53 and be displaced by SAHA/DS treatment, allowing p300 to better interact with it and increase p53 acetylation. We found that this was the case, as SAHA/DS treatment reduced the SET/TAF-Iβ binding to mutp53 while increasing its interaction with p300 (Figure 4D). Altogether, these findings suggest that p300-mediated p53 acetylation was facilitated by SET/TAF-Iβ displacement from mutp53 in SAHA/DS-treated cells.

### 3.3. Valemetostat–SAHA, Even More in Combination with 5-AZA, Impairs Pancreatic Cancer Cell Survival, Reducing CHK1 and Increasing DNA Damage

**Figure 5:**
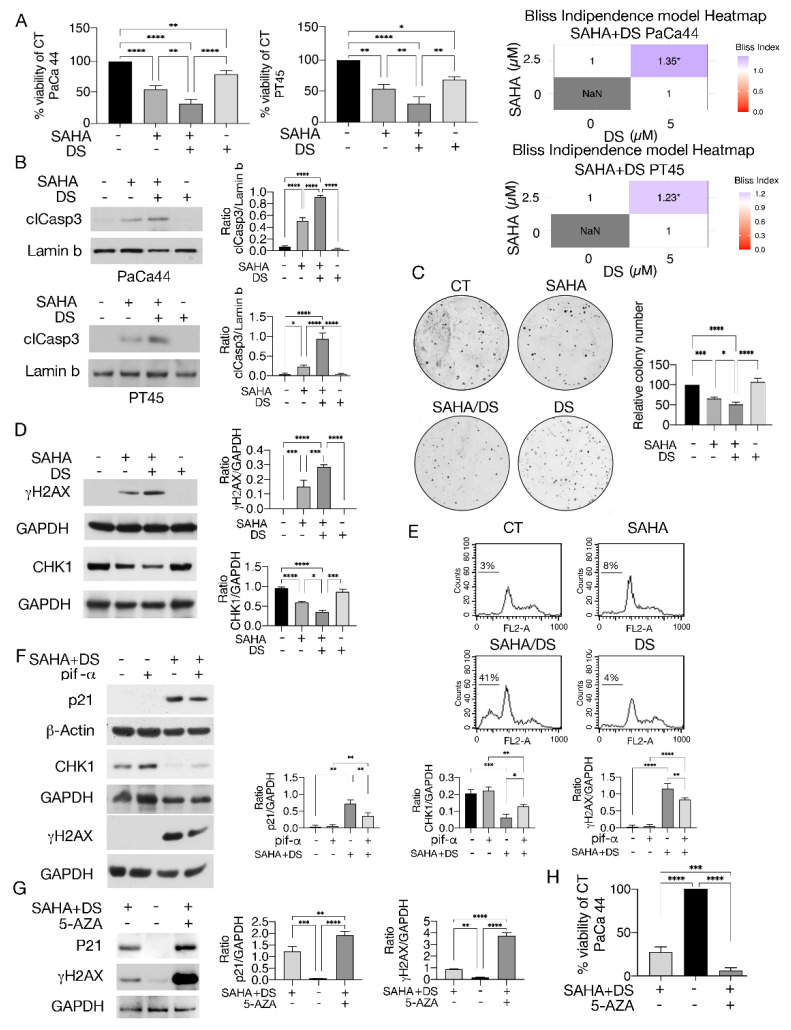
SAHA/DS, particularly with 5-AZA supplementation, reduces pancreatic cancer cell survival, downregulates CHK1, and increases DNA damage in pancreatic cancer cells. (**A**) Cell survival as evaluated by Trypan blue exclusion assay in PaCa44 and PT45 cell lines, untreated (CT) or treated with SAHA, SAHA/DS, or DS. The histograms indicate the percentage of cell viability relative to the control; data are shown as the mean plus SD from more than three experiments. * *p*-value < 0.05; ** *p* < 0.01 and **** *p* < 0.0001 as calculated by ANOVA test. In addition, the synergistic cytotoxicity induced by combination treatment, as evaluated by the Bliss independence model, is reported (Bliss index > 1). (**B**) Cleaved Caspase 3 (cl Casp3) expression level as investigated by Western blotting in PaCa44 and PT45 cells undergoing the above-reported treatments. Lamin b was used as the loading control. Histograms are the mean plus SD of the densitometric analysis carried out in three experiments, expressing the ratio between cleaved Caspase3 and lamin b; * *p*-value < 0.05 and **** *p* < 0.0001 as calculated by ANOVA test. (**C**) Representative pictures of PaCa44 cell colonies following staining with crystal violet and histograms representing the quantitative analyses of colony formation shown as mean ± SD of percent on untreated cells (CT). (**D**) γH2AX and CHK1 expression as evaluated by Western blotting analysis in PaCa44 cells untreated (CT) or treated by SAHA, SAHA/DS, and DS. GAPDH represented the loading control. (**E**) FACS profiles of Paca44 cells treated as reported above. The numbers indicate the percentage of subG1 events. One experiment out of three is shown. (**F**) p21, CHK1, and γH2AX expression as evaluated by Western blotting in PaCa44 cells pre-treated (+) or not (−) with pifithrin-α and exposed to SAHA/DS or left untreated (CT). GAPDH was the loading control. (**G**) p21, CHK1, and γH2AX expression as evaluated by Western blotting in PaCa44 cells untreated (CT) or treated by SAHA/DS in the presence or absence of 5-AZA. GAPDH was the loading control. (**H**) Cell survival as evaluated by Trypan blue exclusion assay in PaCa44 cell lines treated by SAHA/DS in the presence or absence of 5-AZA or left untreated. Histograms represent the mean plus SD of the densitometric analysis derived from three experiments and expressed as the ratio between (**B**) cl Casp3/lamin b, (**D**) γH2AX/GAPDH and CHK1/GAPDH, (**F**) p21/GAPDH, CHK1/GAPDH, and γH2AX/GAPDH, and (**G**) p21/GAPDH and γH2AX/GAPDH. * *p*-value < 0.05; ** *p* < 0.01; *** *p* < 0.001; and **** *p* < 0.0001 as calculated by ANOVA test.

We next analyzed the impact of SAHA, DS, or their combination on pancreatic cancer cell survival. As shown in Figure 5A, drug combination induced a synergistic cytotoxic effect (Bliss index > 1) compared to single treatments, increased caspase-3 cleavage (Figure 5B), and further reduced colony formation (Figure 5C). Additionally, stronger DNA damage was observed, as γH2AX expression levels increased in SAHA/DS-treated cells (Figure 5D). This correlated with the downregulation of CHK1 (Figure 5D), a molecule that plays a key role in the regulation of cell cycle checkpoints and DNA repair [28]. Accordingly, a G1 phase cell cycle arrest was observed in SAHA and SAHA/DS treated cells. Furthermore, the combined treatment induced an increase in subG1 events, indicating the occurrence of apoptotic cell death (Figure 5E). The wtp53-p21 axis has been reported to be implicated in CHK1 downregulation [29], therefore, here we investigated whether this axis could contribute to the reduction in CHK1 by SAHA/DS. For this purpose, we used pifithrin-α and found that in addition to reducing p21, it partially counteracted CHK1 downregulation and mitigated DNA damage (Figure 5F), similar to the study reported above.

In addition to histone modifications, gene expression can be affected by changes in DNA methylation/demethylation. We previously reported that SAHA, combined with the DNA-demethylating agent 5-AZA, enhanced p53 acetylation, upregulated p21, and impaired the survival of pancreatic cancer cells by upregulating metallothionein 2, which can sequester zinc ions required for the activity of zinc-dependent HDACs [8]. In this study, we found that 5-AZA supplementation further increased the p21 expression level and DNA damage in SAHA/DS-treated cells (Figure 5G) and observed that the cytotoxic effect was also enhanced (Figure 5H). These findings support the emerging hypothesis that targeting multiple epigenetic pathways may represent a promising avenue, particularly against solid cancers, because tumor suppressors can be epigenetically silenced through different mechanisms and because single epigenetic treatments can cause rebound epigenetic effects.

## 4. Discussion

Mutations in the *p53* gene can give rise to proteins that sustain oncogenic pathway activation, making targeting of mutant p53 a critical step in cancer therapy. It is emerging that PTMs, particularly acetylation, may represent a promising approach to reduce mutp53 stability and downregulate its expression level [9]. In this study, we demonstrate that the combination of Valemetostat and SAHA led to a reduction in the mutp53 expression level and increased p53 acetylation. Moreover, such treatment enhanced the binding of p53 to the *p21* promoter and the upregulation of this protein, increasing DNA damage in pancreatic cancer cells. At the molecular level, SAHA/DS displaced SET/TAF-Iβ and enhanced the interaction of mutp53 with p300, responsible for p53 acetylation [17]. It has been previously shown that acetylation–methylation cross-talk occurs on histones and other non-histone proteins. For instance, an antagonistic relationship between these PTMs has been observed on histone H3 at the H3K27 residue, where the methylation mediated by PRC2 interferes with p300- and CBP-induced acetylation [30]. Interestingly, the steady-state balance between histone acetylation and methylation has been shown to function as a molecular rheostat in regulating cellular transcription [31]. Regarding non-histone proteins, for example, the cardiac transcription factor GATA4 methylation induced by EZH2 has been reported to prevent p300-mediated acetylation [32].

A complex interplay between methylation and acetylation may also regulate the function of wtp53 [11]. Indeed, p300- and PCAF-induced wtp53 acetylation can be inhibited by SET/TAF-Iβ, resulting in a reduction in its ability to induce cell cycle arrest and apoptosis [17]. Furthermore, the acidic domain of SET, present in almost all enzymes with methyltransferase activity, fails to interact with proteins such as Ku70, FOXO1, and H3 when acetylated, while SET depletion increases p300/CBP-mediated H3K27 acetylation [27]. Although an acetylation–methylation interaction has been previously observed, the present study shows for the first time that a Valemetostat–SAHA combination reduced the binding of SET/TAF-Iβ to mutp53, increased its interaction with p300, and enhanced p53 acetylation. These drugs lead to the downregulation of mutp53 and the activation of *p21* transcription known to regulate the cell cycle, apoptosis, and transcriptional regulation after DNA damage [33], impairing pancreatic cancer cell proliferation. Our findings, together with a previous study reporting that EZH2 supports mutp53 by enhancing its translation [34], strengthen the idea that EZH2 inhibition may help target mutp53 and compromise the cell survival of tumor cells harboring this oncogenic protein. This is particularly relevant in the context of pancreatic cancer, as it is a highly aggressive cancer and resistant to current therapies, although the doses of Valemetostat used in this study are quite high and could cause side effects. This study also supports the emerging hypothesis that epigenetic targeting of multiple biological pathways could be an effective strategy for treating aggressive tumors, as targeting a single epigenetic pathway may inadvertently activate resistance mechanisms [35]. For instance, it has been reported that H3K27 acetylation can increase as a resistance mechanism in response to EZH2 inhibitor treatment in other cancer cells [36]. On the other hand, co-targeting of the epigenetic regulators EZH2 and HDACs has been shown to be more cytotoxic than either treatment alone against castration-resistant prostate cancer [37]. To further support the hypothesis that epigenetic drug combinations can be exploited to improve the outcome of anti-cancer treatment, here we demonstrated that the cytotoxicity of SAHA/DS was enhanced by 5-AZA supplementation, which more strongly upregulates p21, and potentiated DNA damage and cell death in pancreatic cancer cells. As for the molecular mechanism/s, based on our previous study, it is possible that 5-AZA reduced the deacetylating activity of HDAC by downregulating metallothionein 2 (MT2), a protein that sequesters the zinc required for HDAC activity, and through this mechanism could potentiate SAHA activity, further upregulating p21 when added to SAHA/DS in pancreatic cells. However, demethylating activity on the *p21*, *TP53*, or *TP73* promoters could also contribute to p21 upregulation.

## 5. Conclusions

**Figure 6:**
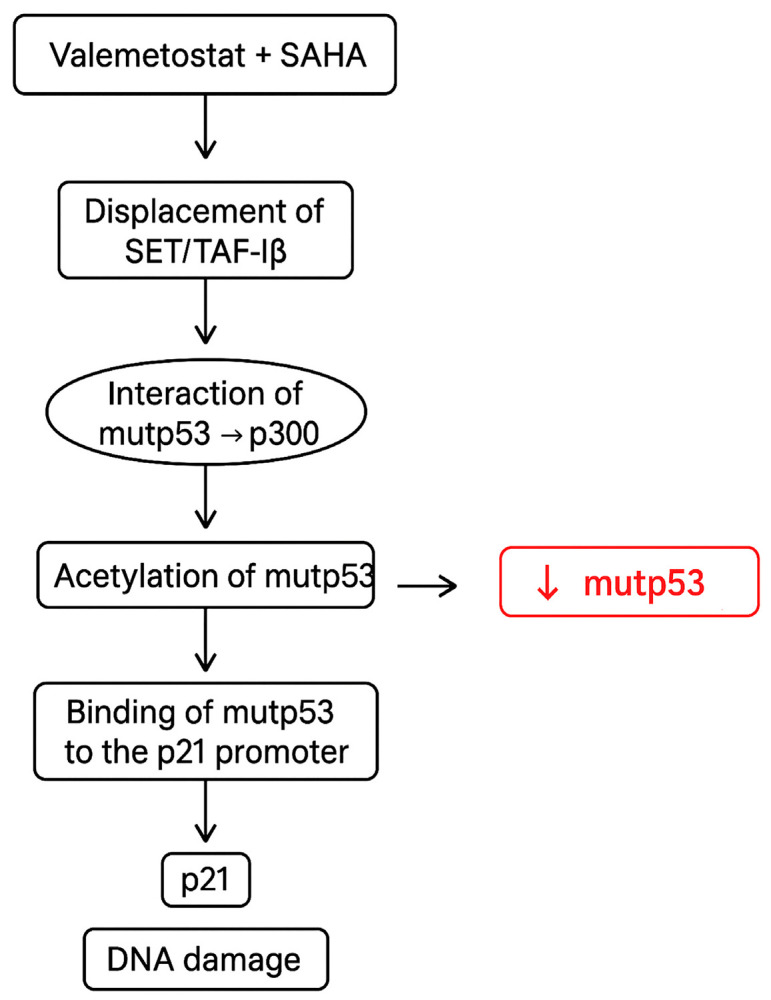
Diagram representing the impact of SAHA/DS treatment on mutp53, p21, and DNA damage in pancreatic cancer cells.

In conclusion, this study demonstrates that the combination of epigenetic drugs such as DS and SAHA increases the interaction of mutp53 with p300, enhancing p53 acetylation. Moreover, this combined treatment downregulates mutp53 and promotes the upregulation of p21, a protein able to regulate the cell cycle and apoptosis and increase DNA damage (Figure 6). This latter effect could be related to the reactivation of wtp53 properties or the acquisition of new functions by mutp53, such as the ability to recruit p63 or p73 to the *p21* promoter. The findings that SAHA/DS induced a stronger cell cycle arrest and apoptosis in pancreatic cancer cells, further enhanced by 5-AZA supplementation, strengthen the hypothesis that targeting multiple epigenetic pathways could be promising against tumors harboring p53 mutations.

## Data Availability

The datasets generated and analyzed during the current study are available from the corresponding author upon reasonable request.
