# Correction: Di Crosta et al. Valemetostat–SAHA-Driven Acetylation of p53 via SET/TAF-Iβ Displacement and p300 Activation Modulates Cell Cycle Regulators in Pancreatic Cancer Cells. *Biomedicines* 2025, *13*, 2279

**DOI:** 10.3390/biomedicines14051159

**Published:** 2026-05-20

**Authors:** Michele Di Crosta, Francesca Chiara Ragone, Rossella Benedetti, Gabriella D’Orazi, Roberta Santarelli, Maria Saveria Gilardini Montani, Mara Cirone

**Affiliations:** 1 Department of Experimental Medicine, Sapienza University of Rome, 00161 Rome, Italy; michele.dicrosta@uniroma1.it (M.D.C.); ragone.1872024@studenti.uniroma1.it (F.C.R.); rossella.benedetti@uniroma1.it (R.B.); roberta.santarelli@uniroma1.it (R.S.); 2 Faculty of Medicine, UniCamillus—Saint Camillus International University of Health and Medical Sciences, 00131 Rome, Italy; gabriella.dorazi@unicamillus.org; 3 Unit of Cellular Networks and Molecular Therapeutic Targets, IRCCS Regina Elena National Cancer Institute, 00144 Rome, Italy

In the original publication [1], there was a mistake in Figure 5 as published.

The mistake consists of the overlap between the lane corresponding to Lamin b for PT45 cells in Figure 5B and the lane corresponding to GADPH (p21) for PT45 cells in Figure 4A, taken from a previously published paper by the same authors [2].

The corrected Figure 5 appears below. The authors state that the scientific conclusions are unaffected. This correction was approved by the Academic Editor. The original publication has also been updated.

## Figures and Tables

**Figure 5:**
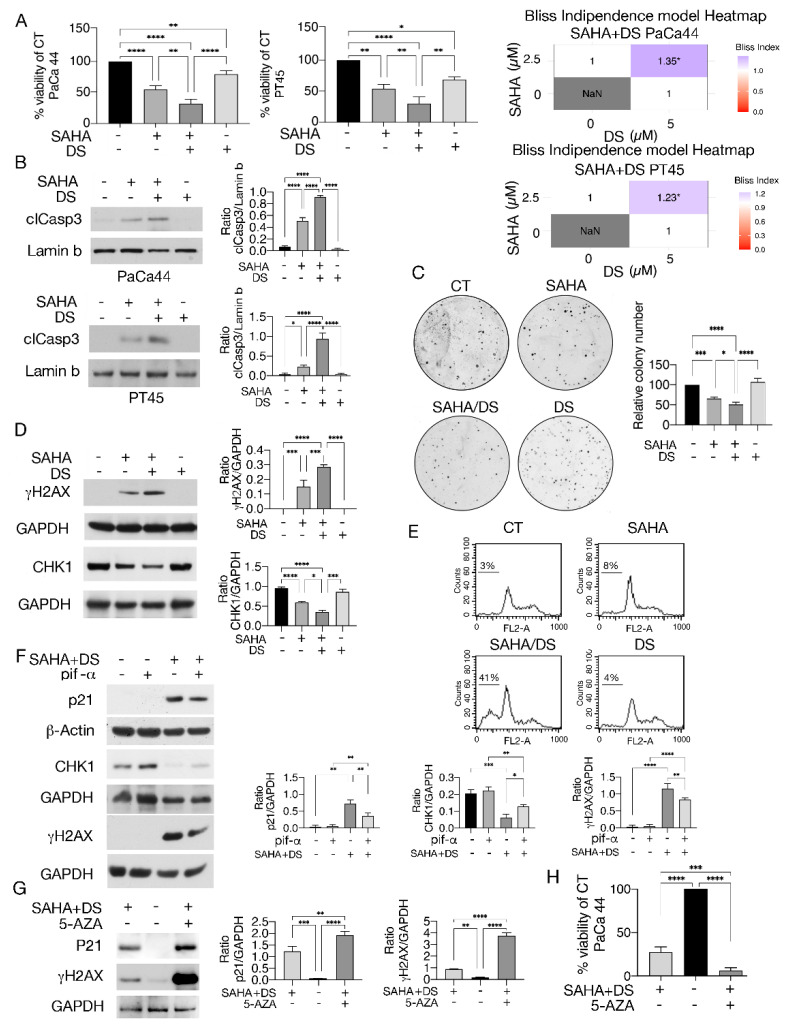
SAHA/DS, particularly with 5-AZA supplementation, reduces pancreatic cancer cell survival, downregulates CHK1, and increases DNA damage in pancreatic cancer cells. (**A**) Cell survival as evaluated by Trypan blue exclusion assay in PaCa44 and PT45 cell lines, untreated (CT) or treated with SAHA, SAHA/DS, or DS. The histograms indicate the percentage of cell viability relative to the control; data are shown as the mean plus SD from more than three experiments. * *p*-value < 0.05; ** *p* < 0.01 and **** *p* < 0.0001 as calculated by ANOVA test. In addition, the synergistic cytotoxicity induced by combination treatment, as evaluated by the Bliss independence model, is reported (Bliss index > 1). (**B**) Cleaved Caspase 3 (cl Casp3) expression level as investigated by Western blotting in PaCa44 and PT45 cells undergoing the above-reported treatments. Lamin b was used as the loading control. Histograms are the mean plus SD of the densitometric analysis carried out in three experiments, expressing the ratio between cleaved Caspase3 and lamin b; * *p*-value < 0.05 and **** *p* < 0.0001 as calculated by ANOVA test. (**C**) Representative pictures of PaCa44 cell colonies following staining with crystal violet and histograms representing the quantitative analyses of colony formation shown as mean ± SD of percent on untreated cells (CT). (**D**) γH2AX and CHK1 expression as evaluated by Western blotting analysis in PaCa44 cells untreated (CT) or treated by SAHA, SAHA/DS, and DS. GAPDH represented the loading control. (**E**) FACS profiles of Paca44 cells treated as reported above. The numbers indicate the percentage of subG1 events. One experiment out of three is shown. (**F**) p21, CHK1, and γH2AX expression as evaluated by Western blotting in PaCa44 cells pre-treated (+) or not (−) with pifithrin-α and exposed to SAHA/DS or left untreated (CT). GAPDH was the loading control. (**G**) p21, CHK1, and γH2AX expression as evaluated by Western blotting in PaCa44 cells untreated (CT) or treated by SAHA/DS in the presence or absence of 5-AZA. GAPDH was the loading control. (**H**) Cell survival as evaluated by Trypan blue exclusion assay in PaCa44 cell lines treated by SAHA/DS in the presence or absence of 5-AZA or left untreated. Histograms represent the mean plus SD of the densitometric analysis derived from three experiments and expressed as the ratio between (**B**) cl Casp3/lamin b, (**D**) γH2AX/GAPDH and CHK1/GAPDH, (**F**) p21/GAPDH, CHK1/GAPDH, and γH2AX/GAPDH, and (**G**) p21/GAPDH and γH2AX/GAPDH. * *p*-value < 0.05; ** *p* < 0.01; *** *p* < 0.001; and **** *p* < 0.0001 as calculated by ANOVA test.
